# Contribution of migratory types to the reproduction of migrating silver eels in a tropical eel, *Anguilla bicolorbicolor*

**DOI:** 10.1016/j.heliyon.2022.e09491

**Published:** 2022-05-20

**Authors:** Takaomi Arai, Naoko Chino

**Affiliations:** a Environmental and Life Sciences Programme, Faculty of Science, Universiti Brunei Darussalam, Jalan Tungku Link, Gadong, BE 1410, Brunei Darussalam; b Atmosphere and Ocean Research Institute, The University of Tokyo, 5-1-5, Kashiwanoha, Kashiwa, Chiba 277-8564 Japan

**Keywords:** Anguillid eel, Catadromous, Life history, Migration, Reproduction, Tropical eel

## Abstract

The recent noticeable depletion of the catadromous eel populations has created global concerns for the eel stocks. Thus, the demand for tropical catadromous eels, which are a major alternate target species to compensate temperate species, is sharply increasing. However, eminently little is known regarding the biology and ecology of tropical eels across the Indo-Pacific region. To elucidate the contribution to reproduction among migratory types in a tropical eel, *Anguilla bicolor bicolor*, which is currently considered a major commercial target species, the otolith microchemistry was examined in silver (matured) stage eels. The broad range of otolith Sr:Ca ratios indicated that the habitat use was opportunistic among sea, brackish and fresh waters after recruitment to the continental habitats. Two migratory types, estuarine resident and marine resident eels, were found, but no freshwater resident eel, which is considered as the typical catadromous migration, was found. The lack of freshwater resident eels among all silver eels suggests that the estuarine and marine migrants colonizing adjacent to coastal areas might facilitate a substantial contribution to reproduction for the following generation in this area.

## 1 Introduction

Catadromous eels in the genus *Anguilla* are biologically important animals because they have a unique life history and are economically important because they are used as food resources. These eels have attracted scientists over long periods of time because of the remarkable catadromous migration between continental growth habitats and open ocean spawning grounds as well as their complex ecology. American, European and Japanese eel populations now are considered to be outside the safe biological limits and are severely menaced by extinction [1, 2, 3]. Therefore, these eels are also important animals from a conservation standpoint.

Nineteen species of catadromous eels have been found throughout the world, 13 of which are reported from tropical areas [4, 5]. Of those tropical species, seven species were found around Indonesia in the western Pacific [4, 5]. Tropical eels are believed to be originated in the Indonesian region as the most basal species and thereafter the catadromous eels dispersed to the temperate regions from the tropics [6, 7]. Therefore, tropical eels could be more firmly associated to the ancestor of eels than temperate species. To understand the origin of the ancestral catadromous migration, it is crucial to study the biology and ecology of tropical eels. The recent serious depletion in recruitment of glass eels (juveniles) in European and East Asian countries has created severe issues in adult eel population and stock in natural environments [1, 2, 3]. Tropical eels, especially *Anguilla bicolor* (*A. bicolor bicolor* and *A. bicolor pacifica*), are currently major targets to fulfill the high requirement to compensate for the declined temperate eel resources [1, 3]. However, the life history, spawning ecology and spawning area of the tropical eels in the Indo-Pacific region are extremely unknown [6, 8, 9].

Otolith microchemistry research indicated that yellow (immature) and silver (mature) eels lived their whole life history in the coastal area (marine residents) without the typical freshwater life history (freshwater residents) [10]. Research on otolith strontium (Sr):calcium (Ca) ratios has also elucidated the alternative migratory histories which is called “estuarine residents” shifting between different salinity environments [10]. Therefore, freshwater life are not absolutely necessary for all catadromous eels and that the eels exhibit opportunistic catadromous migration [11].

Although otolith microchemistry in some temperate and tropical catadromous eels have been examined [10], there have been a few studies of migratory history using silver stage eels which has longer migratory history than that of yellow stage eels. Therefore, it is not clear how each eel utilizes both estuarine and marine environments besides the freshwater environments that are typically used throughout their entire lives. Furthermore, because silver eels are difficult to collect in the coastal waters and open ocean spawning area, there is little known about the relative contribution to reproduction in each migratory type.

In order to elucidate this question, otolith microchemistry in silver eels of a tropical catadromous eel *A. bicolor bicolor* that started their oceanic spawning migration, were analyzed. To understand the diverse migratory history in the tropical catadromous eel, otolith Sr:Ca ratios were examined in silver eels throughout the year. We estimated the possible contribution to reproduction in each migratory type, i.e., marine residents, estuarine residents and freshwater residents.

## 2 Materials and methods

### 2.1 Eel samples

**Figure 1:**
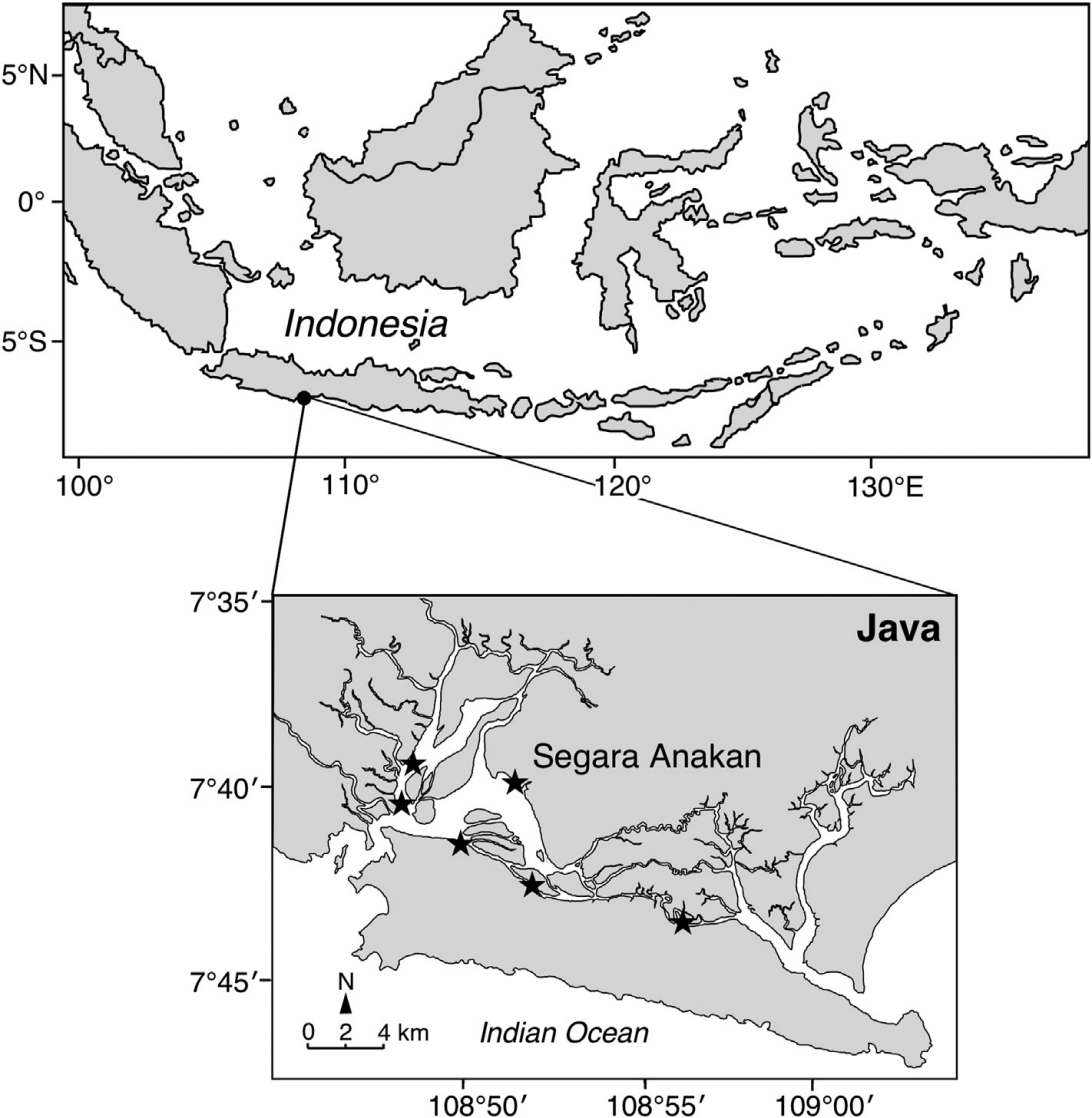
Locations where tropical catadromous eels were collected. A map showing the location of Segara Anakan, central Java Island, Indonesia. The sampling site where *Anguilla bicolor bicolor* specimens were collected in the area are displayed with small stars. The map was drawn by the author using Adobe Illustrator CS6 and referring to Google Maps 2021 (https://maps.google.com/).

A total of 287 *Anguilla bicolor bicolor* were collected in Segara Anakan Lagoon of Cilacap Regency, south coast of west and central Java, Indonesia (7°35′–7°48 S, 108°46–109°03′E) from December 2007 to January 2009 (Figure 1). The water temperature and salinity fluctuated between 27.5 °C (dry season) and 35.0 °C (wet season) and between 8 to 28 ppt (dry season) and 1–20 ppt (wet season), respectively [12, 13]. The eels were caught using bamboo traps and angling. All specimens were purchased from local fishermen and thereafter frozen immediately. Our protocols followed the ethical guidelines for the use of animals of Universiti Brunei Darussalam (UBD) and were approved by the animal ethics committee at UBD.

The total length (TL) and body weight (BW) were measured and the sex and maturation stage of each eel was determined by histological observations of the gonads [8, 14]. Eels in more advanced maturation stages (silver stage), such as stages IV (vitellogenic oocytes) and V (midvitellogenic oocytes), were selected in the present study. A total of 60 silver stage eels, including 27 eels in stage IV and 33 eels in stage V, were used for further otolith microchemical analysis. All eels were females.

### 2.2 Life-history transect analysis in otolith

Sagittal otoliths were extracted from each fish, embedded in epoxy resin (Struers, Epofix), mounted on glass slides and the otoliths were ground and polished [15]. Life-history transect analysis of Sr and Ca concentrations in all otoliths were conducted by means of a wavelength dispersive X–ray electron microprobe (JEOL JXA-8900R) [16, 17]. The beam current and accelerating voltage were 1.2 × 10^−8^ A and 15 kV, respectively and Wollastonite (CaSiO_3_) and Tausonite (SrTiO_3_) were used as standards. The measurements spaced at 10 μm intervals and the electron beam was focused on a point 10 μm in diameter. The Sr and Ca counting times at their peaks were both 10 s.

The average Sr:Ca ratios outside the elver mark were calculated, the eel habitats were categorized into “marine” (Sr:Ca ≥ 6.0 × 10^−3^), “estuarine” (2.0 × 10^−3^ ≤ Sr:Ca < 6.0 × 10^−3^) and “freshwater” (Sr:Ca < 2.0 × 10^−3^) according to the criteria in tropical anguillid eels [12, 13, 15, 16, 17, 18].

### 2.3 Statistical analysis

The differences in TL and BW between stage IV and stage V were examined through a Mann-Whitney-*U* test. The differences between the low and high Sr:Ca ratio phases along the life history transect of each otolith were examined through a Mann-Whitney-*U* test.

## 3 Results

### 3.1 Eel characteristics

TL of *Anguilla bicolor bicolor* ranged from 557 to 803 mm, and BW ranged from 220 to 903 g (Table 1). There were no significant differences in TL and BW between stage IV and stage V in the silver stages (Mann-Whitney *U*-test, p > 0.5). The mean ± SD of TL and BW for all specimens was 641 ± 54.3 mm and 463 ± 138 g, respectively.

**Table 1:** Silver stage eels of *Anguilla bicolor bicolor* used in this study.

Month	Number of eels	Total length (mm)		Body weight (g)	
		Range	Mean	Range	Mean
December 2007	2	557–649	-	220–442	-
March 2008	2	565–635	-	285–507	-
May 2008	14	568–744	625 ± 55.5	300–734	463 ± 128
August 2008	7	652–686	669 ± 11.1	475–609	537 ± 52.8
September 2008	18	568–741	635 ± 48.5	257–713	413 ± 126
October 2008	5	590–781	662 ± 80.1	322–783	505 ± 178
November 2008	2	568–664	-	344–585	-
December 2008	5	610–688	663 ± 31.1	438–677	537 ± 95.5
January 2009	5	589–803	669 ± 81.2	345–903	502 ± 227

#### 3.1.1 Life history transects

**Figure 2:**
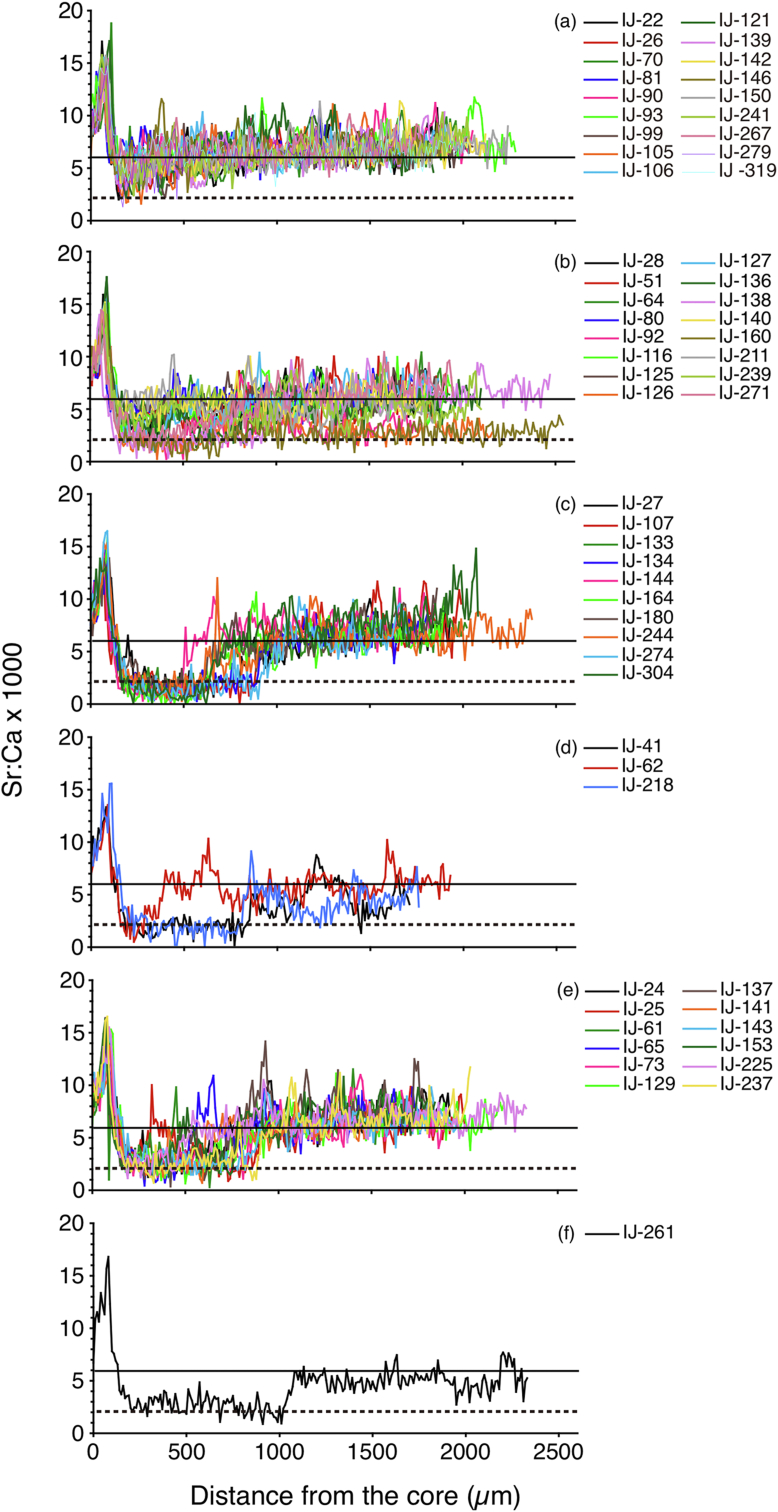
Migratory histories of the silvering stage of a tropical catadromous eel *Anguilla bicolor bicolor*. Sr:Ca variations along line transects from the otolith core to the edge for all specimens (60). Eels showing a resident pattern lived in either marine water (a) or brackish water (b) across their lives. Eels showing a migrant pattern moved between those habitats. The variations included eels that migrated from freshwater to marine water (c), from freshwater to brackish water (d), from brackish water to marine water (e), and from lower brackish water to higher brackish water (f). The solid line in each panel indicates marine water life period (≥6.0 × 10^−3^ in Sr:Ca ratios), and the dotted line in each panel indicates freshwater life period (<2.0 × 10^−3^ in Sr:Ca ratios). Numbers on the upper right indicate fish number.

Changes in otolith Sr:Ca ratios along line transects showed a common trait in all specimens from the otolith core to approximately 150 μm showing a peak of high Sr:Ca ratios (12.5 × 10^−3^ – 18.8 × 10^−3^) (Figure 2). This region coincides with oceanic migration life during leptocephalus (larval) stage [10]. Leptocephalus accumulates significant amounts of gelatinous extracellular material in the body before metamorphosis to glass eel (juvenile) [19].

There were classified into two types in the otolith Sr:Ca ratios outside the high Sr:Ca rations around the core region (Figure 2). The first pattern showed the resident type, and it has comparatively constant Sr:Ca ratios (range: 2.88–7.10 × 10^−3^; mean ± SD 5.81 × 10^−3^ ± 1.05 × 10^−3^) over the life history transect. These specimens are suggested to reside in the same salinity level over their lives (Figure 2a, b n = 34, 56.7 %). These eels could be further categorized into two groups. Eighteen of the 34 samples had higher Sr:Ca ratios (range: 6.00–7.10 × 10^−3^; mean ± SD 6.48 × 10^−3^ ± 0.33 × 10^−3^) (Figure 2a), whereas 16 of the 34 samples had consistently middle-range Sr:Ca ratios (range: 2.88–5.93 × 10^−3^; mean ± SD 5.00 × 10^−3^ ± 1.06 × 10^−3^) (Figure 2b) throughout the otolith. The constant eels with Sr:Ca ratios greater than 6.0 × 10^−3^ and consistently middle-range Sr:Ca ratios between 2.0 and 6.0 could be labelled marine residents and estuarine residents, respectively.

The second pattern showed the migrant type which shifted between the inner and outer parts of the otolith Sr:Ca ratios along the transect. The migrant type could be further categorized into four groups. The Sr:Ca ratios in the inner part were low (range: 0.87–1.96 × 10^−3^, mean ± SD 1.61 × 10^−3^ ± 0.39 × 10^−3^) and thereafter increased to a high level (range: 6.19–7.73 × 10^−3^; mean ± SD 6.68 × 10^−3^ ± 0.55 × 10^−3^) in the first group (Figure 2c, n = 10). The Sr:Ca ratios in the inner part were low (range: 1.89–1.93 × 10^−3^; mean ± SD 1.90 × 10^−3^ ± 0.02 × 10^−3^) and then increased to a middle range (range: 4.58–5.77 × 10^−3^; mean ± SD 4.98 × 10^−3^ ± 0.68 × 10^−3^) in the second group (Figure 2d, n = 3). The Sr:Ca ratios in the inner portion were intermediate (range: 2.36–3.61 × 10^−3^; mean ± SD 2.83 × 10^−3^ ± 0.40 × 10^−3^) and thereafter increased to a high level in the outer part of the transect in the third group (range: 6.07–7.04 × 10^−3^; mean ± SD 6.63 × 10^−3^ ± 0.29 × 10^−3^) (Figure 2e, n = 12). The Sr:Ca ratios in the inner part were a lower intermediate level (2.36 × 10^−3^) and thereafter increased to a higher intermediate level but were less than 6.0 × 10^−3^ in the outer portion of the transect (5.02 × 10^−3^) in the fourth group (Figure 2f, n = 1). The transition occurred between 280 and 1010 μm (mean ± SD 705 ± 178 μm) from the otolith core in all migrant eels. These transitions suggest that these eels shifted their habitat and migrated to a higher salinity habitat (Figure 2c, d, e, f; n = 26). Overall, 43.3 % of eels exhibited the migrant type. Significant differences were found between the low and high phases in otolith Sr:Ca ratios for all migrant type eels (Mann-Whitney *U*-test p < 0.0001). We could label the migrant type eels from group 1 to group 4 as habitat shifting from freshwater to seawater, habitat shifting from fresh water to brackish water, habitat shifting from brackish water to seawater and habitat shifting from low brackish water to high brackish water, respectively.

### 3.2 Habitat use

**Figure 3:**
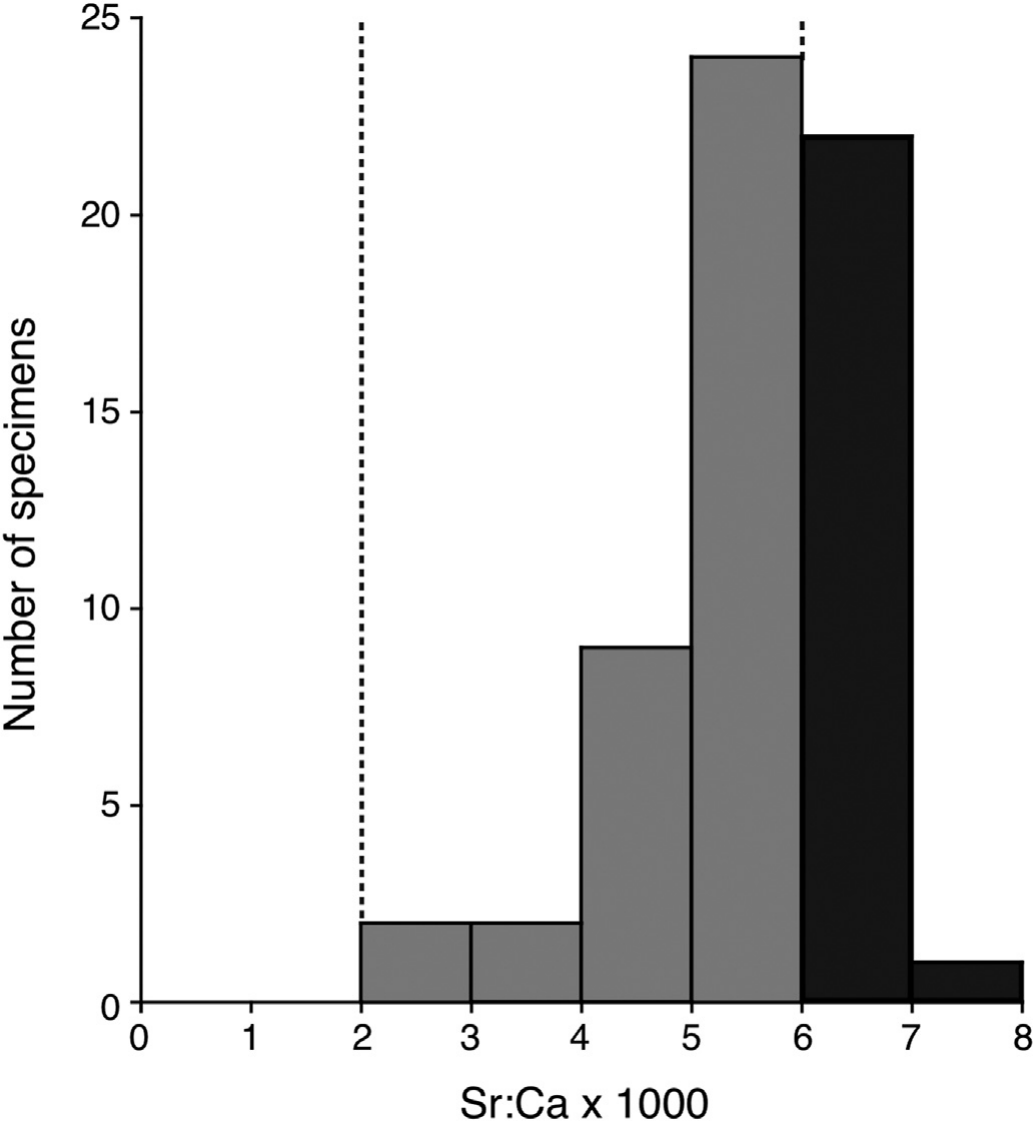
Habitat use of the silvering stage of a tropical catadromous eel *Anguilla bicolor bicolor*. Frequency distribution of mean Sr:Ca ratios (right) in each fish outside the elver mark (150 μm in radius).

The average Sr:Ca ratio during the continental life in each eel ranged from 2.88 to 7.10 × 10^−3^ (Figure 3). The broad range of otolith Sr:Ca ratios suggest that the habitat use was different and variable after recruitment to the continental habitats. Their migratory types were either marine resident (n = 18, 30 %) or estuarine resident (n = 42, 70 %) (Figure 3). However, none of the eels had a typical catadromous migration pattern (less than 2.0 × 10^−3^) in the present study (Figure 3).

**Figure 4:**
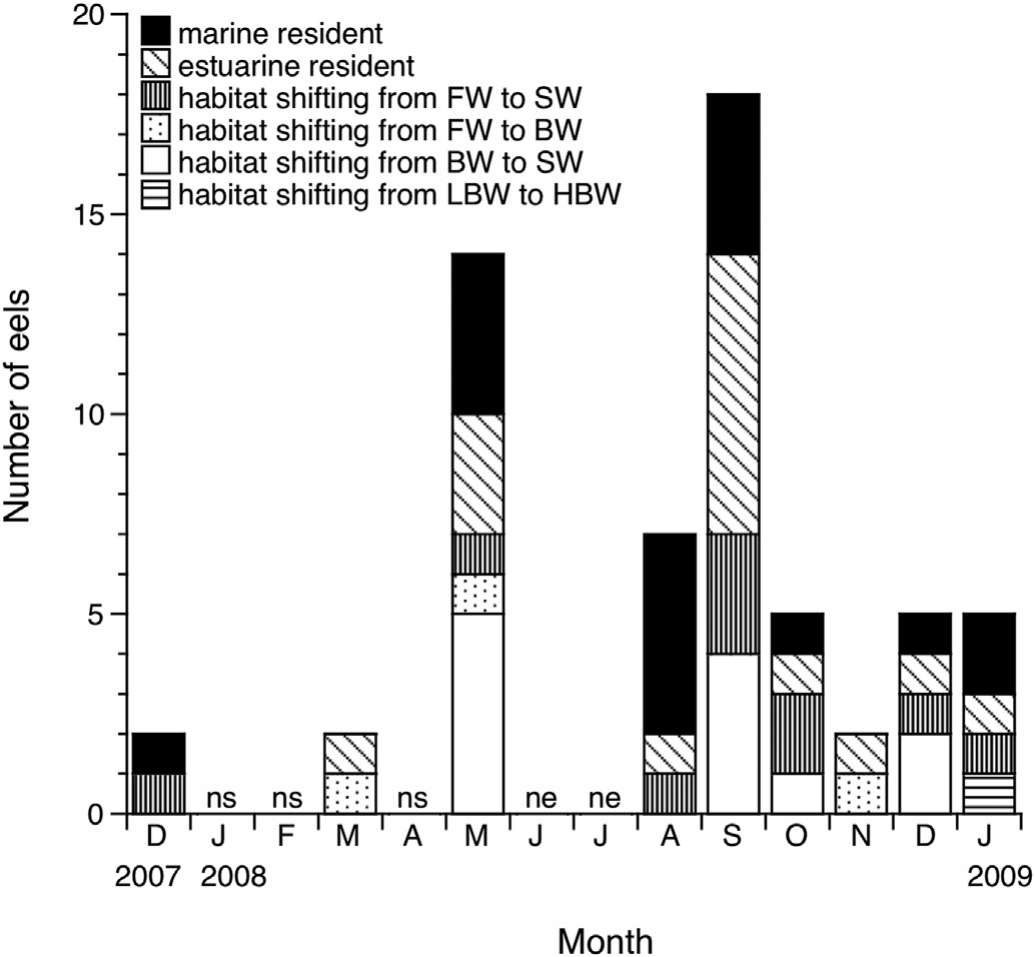
Monthly occurrence in migratory types of the silvering stage of a tropical catadromous eel *Anguilla bicolor bicolor*. Monthly fluctuation of migratory types in a tropical freshwater eel *Anguilla bicolor bicolor* collected in central Java, Indonesia between December 2007 and July 2009. ns: no study for eel sampling was conducted that month. ne: study was conducted, but no eels were collected. FW: freshwater, SW: seawater, BW: brackish water, LBW: lower brackish water, HBW: higher brackish water.

Silver eels were found throughout the year, although the frequency differed among months (Figure 4). There were no dominant monthly occurrences of each migratory type throughout the year. The marine resident, estuarine resident and habitat shifting eels occurred almost every month (Figure 4). However, freshwater resident eels were not found throughout the year, even though all of the eels were in the condition of downstream migration to open ocean.

## 4 Discussion

It is quite remarkable that eels with a typical catadromous pattern (freshwater resident) were not found throughout the year and that only marine and estuarine resident eels occurred in the study area (Figures 2, 3, and 4). The broad range of otolith Sr:Ca ratios suggested that the habitat use of *Anguilla bicolor bicolor* during their continental growth phase just before spawning migration is opportunistic and not obligatory. These findings vigorously indicated that *A. bicolor bicolor* has an elastic life history strategy, with a potency of migratory flexibility and a potential to exploit the broad range of salinity. Among migrating silver eels, such as the Japanese eel (*A. japonica*), collected in the Pacific Ocean off the central part of Japan, the proportion of freshwater residents was the lowest (19 %) and marine residents and estuarine residents were plentiful in the region [20]. These results suggest that the estuarine and marine residents that inhabiting adjacent to coastal areas may make a substantial contribution to reproduction to the following generation in catadromous eels.

In the Atlantic eels, considering both European (*A. anguilla*) and American (*Anguilla rostrata*) eels, Tucker [21] hypothesized that European eels could not accomplish the spawning migration from Europe and North Africa to the Sargasso Sea because the distance between their freshwater growth habitats and spawning area is much longer than that of the American eels. Tucker [21] therefore concluded that only American eels can contribute to the reproduction of the Atlantic eel population, which should therefore be genetically homogeneous and constitute one species, although this hypothesis and conclusion were refused by further studies. However, the current study considers not the species level but the ecological population level, suggesting that freshwater resident eels of *A. bicolor bicolor* might make smaller or no contribution to reproduction and that such populations might not undertake downstream migration to the open ocean spawning area. The abiotic and biotic environments underwent in the anguillid eels during the continental growth are found to influence differences in life history and reproductive success [22, 23, 24]. Habitat loss during continental life could threaten the maintenance of eel populations through a loss of reproductive success [25, 26]. Anguillid eels are really susceptible to organic contaminants that influence reproductive failure [27, 28, 29]. Therefore, the eel growth in freshwater environments are more vulnerable to possible effects of organic contaminants on reproduction and survival than that in marine waters [28, 29]. The freshwater resident eel might need to adapt from freshwater to seawater environments via osmoregulation during their downstream migrations. Conversely estuarine resident and marine resident eels might have less or no need to maintain the osmolality and ion concentrations of their body fluid at levels different from the ambient environments during their downstream migrations compared to freshwater resident eels. Regulation of osmolarity of the environmental salinity is a physiological stress encountered across various aquatic organisms. Therefore, the differences in adaptation to varying salinity between freshwater resident and marine and estuarine resident eels during their downstream migration might induce the large differences in reproductive contribution between them.

The wide range of otolith Sr:Ca ratios found in *Anguilla bicolor bicolor* indicated that the tropical catadromous eel had more elastic habitat use, similar to that of other temperate catadromous eels from European, American, Japanese and Australasian (*A. australis* and *A. dieffenbachii*) eels [6, 10, 30]. Landlocked or freshwater resident populations that do not have the oceanic life frequently exist in anadromous salmon, specifically around the southern boundary of their geographical distribution [31]. Anadromous salmons are believed to have originated from a freshwater ancestor, extended their growth habitat into the ocean, while their spawning ground remained in freshwater. In anadromous salmons, reproduction in freshwater was considered to be evolutionary conservative phenomena due to reproduction is physiologically costly [32]. Conversely, the oceanic spawning in the catadromous eels are presumably a conservative phenomenon because the eels are believed to have originated from a marine ancestor [6, 33]. Therefore, a lot of catadromous eels have maintained the potential to live in coastal and marine habitats during the continental life history just before initiating their oceanic migration to the spawning area.

Migration in diadromous fish between freshwater and marine habitats is typically elucidated by a variability in aquatic productivity [34]. In anadromous salmons, after the juvenile salmon emerge from the redds in low productivity freshwater habitas at high latitudes, they immigrate into the ocean with higher productivity for preferable growth prior to returning to their freshwater habitats for reproduction. Conversely, spawning areas in catadromous eels are all located in tropical ocean with low-productivity and hence the eels may immigrate into freshwater habitats with higher productivity for preferable growth. However, all *A. bicolor bicolor* examined in the present study were marine or estuarine resident eels without typical freshwater resident eels in the tropical region near the equator. Therefore, the hypothesis of latitudinal cline in migration patterns in the catadromous eels that the occurrence of freshwater resident eels is predicted higher at low latitudes, where the aquatic productivity in freshwater is higher than that in the ocean [35], may not be supported.

Both estuarine resident and marine resident eels initiates their oceanic migration for breeding at a similar occasion [13]. The synchronized gonadal maturation and migration, and the predominance of estuarine and marine migrants in both this study in a tropical eel and a temperate eel of *A. japonica* [20] have crucial indications for the management and conservation of the catadromous eels. These findings suggest that eels from both estuarine and marine residents can mingle together during the oceanic migration for spawning and possibly promote to the following generation. Thus, estuarine and coastal habitats may be crucial for the catadromous eels in the world.

## Declarations

### Author contribution statement

Takaomi Arai: Conceived and designed the experiments; Analyzed and interpreted the data; Contributed reagents, materials, analysis tools or data; Wrote the paper.

Naoko Chino: Performed the experiments.

### Funding statement

This work was supported by Universiti Brunei Darussalam under the Competitive Research Grant Scheme (UBD/OVACRI/CRGWG (003)) and under the Faculty/Institute/Center Research Grant (UBD/RSCH/1.4/FICBF(b)/2019/021, UBD/RSCH/1.4/FICBF(b)/2020/029 and UBD/RSCH/1.4/FICBF(b)/2021/037).

### Data availability statement

Data will be made available on request.

### Declaration of interests statement

The authors declare no conflict of interest.

### Additional information

No additional information is available for this paper.
